# Proton Magnetic Resonance Spectroscopy in Adults with Childhood Lead Exposure

**DOI:** 10.1289/ehp.1002176

**Published:** 2010-10-13

**Authors:** Kim M. Cecil, Kim N. Dietrich, Mekibib Altaye, John C. Egelhoff, Diana M. Lindquist, Christopher J. Brubaker, Bruce P. Lanphear

**Affiliations:** 1 Cincinnati Children’s Environmental Health Center at Cincinnati Children’s Hospital Medical Center, Cincinnati, Ohio, USA; Departments of; 2 Radiology; 3 Pediatrics; 4 Environmental Health, University of Cincinnati College of Medicine, Cincinnati, Ohio, USA; 5 Department of Psychiatry and Human Behavior, Brown University, Providence, Rhode Island, USA; 6 Child and Family Research Institute, BC Children’s Hospital, Faculty of Health Sciences, Simon Fraser University, Vancouver, British Columbia, Canada

**Keywords:** basal ganglia, brain, cerebellum, frontal lobe, gray matter, lead exposure, magnetic resonance spectroscopy, white matter

## Abstract

**Background:**

Childhood lead exposure adversely affects neurodevelopment. However, few studies have examined changes in human brain metabolism that may underlie known adverse cognitive and behavioral outcomes.

**Objective:**

We examined the association between mean childhood blood lead levels and *in vivo* brain metabolite concentrations as adults, determined by proton magnetic resonance spectroscopy (MRS) in a birth cohort with documented low-to-moderate lead exposure.

**Methods:**

Adult participants from the Cincinnati Lead Study [*n* = 159; mean age (± SD), 20.8 ± 0.9 years] completed a quantitative, short-echo proton MRS protocol evaluating seven regions to determine brain concentrations of *N*-acetyl aspartate (NAA), creatine and phosphocreatine (Cr), cholines (Cho), myo-inositol, and a composite of glutamate and glutamine (GLX). Correlation and multiple linear regression analyses were conducted.

**Results:**

Mean childhood blood lead levels were associated with regionally specific brain metabolite concentrations adjusted for age at imaging and Full-Scale intelligence quotient. Adjusted analyses estimated for a unit (micrograms per deciliter) increase in mean childhood blood lead concentrations, a decrease of NAA and Cr concentration levels in the basal ganglia, a decrease of NAA and a decrease of Cho concentration levels in the cerebellar hemisphere, a decrease of GLX concentration levels in vermis, a decrease of Cho and a decrease of GLX concentration levels in parietal white matter, and a decrease of Cho concentration levels in frontal white matter.

**Conclusions:**

Gray-matter NAA reductions associated with increasing childhood blood lead levels suggest that sustained childhood lead exposure produces an irreversible pattern of neuronal dysfunction, whereas associated white-matter choline declines indicate a permanent alteration to myelin architecture.

Despite public health efforts toward reducing body lead burden, exposure to lead hazards remains an international environmental health problem (Brown et al. 2005; Graber et al. 2010; Haefliger et al. 2009; Hozhabri et al. 2004; Kaiser et al. 2001; Mayan et al. 2001; Nichani et al. 2006; Olivero-Verbel et al. 2007; Rubin et al. 2002; Safi et al. 2006; Zhang et al. 2009). Lead hazards, including paint in toys and consumer products, contaminants in foods and herbal medicines, and residential contamination from dust, water lines, and soil continue to threaten children’s health (Bryant 2004; Clark et al. 2009; Dixon et al. 2009; Edwards et al. 2009; Gould 2009; Jacobs et al. 2002; Karri et al. 2008; Lin et al. 2010; Sathyanarayana et al. 2006). In the United States, contaminated older housing stock (built before 1950) continues to expose significant numbers of children to elevated lead environments (dust and soil) (Gaitens et al. 2009; Jacobs et al. 2002; Kemp et al. 2007; Meyer et al. 2005, 2008). Thus, improving our understanding of how the brain manages lead exposure throughout life and at all exposure levels continues to be an important area for research.

Cognitive and executive dysfunction, learning disabilities, and antisocial behaviors are among the impairments attributed to lead exposure in children that persist into adulthood (Dietrich et al. 1993b; Needleman and Gatsonis 1990; Needleman et al. 1996; Wright et al. 2008). Lead exposure, even at levels < 10 μg/dL, has been associated with these adverse effects in children (Bellinger et al. 1992; Canfield et al. 2003a, 2004; Chandramouli et al. 2009; Lanphear et al. 2005). Although much is known regarding the neurotoxicity of lead, how lead produces these clinical deficits remains poorly understood. The influence of lead exposure on the developing central nervous system and the mechanisms by which lead disrupts brain metabolism in children are complex (reviewed by Bressler and Goldstein 1991; Finkelstein et al. 1998; Lidsky and Schneider 2003; Toscano and Guilarte 2005; White et al. 2007). Toscano and Guilarte (2005) devised a conceptual framework illustrating the effects of developmental lead exposure at glutamatergic synapses and associated signal pathways. Summarizing data from various experimental models of developmental lead exposure, the evidence supports a reduced number of synaptic *N*-methyl-d-aspartate (NMDA) excitatory amino acid receptors (reviewed by Toscano and Guilarte 2005). Lead may act as a calcium analog in neurons, with exposure inhibiting glutamate release through binding to the NMDA receptor in an age-dependent and region-specific manner, which could produce functional differences despite uniform concentrations within the brain. Thus, different regions of the brain can demonstrate variable amounts of injury despite having equivalent lead exposure.

Clinical neuroimaging studies in children with low to moderate (up to 40 μg/dL) blood lead levels tend to have few specific anatomical findings described as characteristic of lead exposure. Neural changes, such as microscopic structural and chemical disruptions, at levels below the detection limits of conventional imaging modalities such as computed tomography or magnetic resonance imaging (MRI) must exist if lead is indeed the source of cognitive deficits and adverse behavioral outcomes. Therefore, a different *in vivo* imaging approach is needed to determine neurochemical alterations. Proton magnetic resonance spectroscopy (MRS) acquired within an MRI examination provides an *in vivo* measure of brain metabolites such as *N*-acetyl aspartate (NAA), creatine and phosphocreatine (Cr), glycerolphosphocholine and phosphocholines (Cho), myo-inositol (mI), and a composite of glutamate and glutamine (GLX) at concentration levels on the order of 1–10 millimolar (mM) (Cecil and Jones 2001). These brain metabolites reflect the functional status of neural structures, such as neurons, axons, glia, as well as myelin and cellular membrane components; however, measurement of neurotransmitters such as dopamine, serotonin, and others is below the detection threshold.

Two groups have examined childhood lead exposure by employing either one of the two standard MRS acquisition approaches. First, Trope et al. (1998) were the first to apply magnetic resonance spectroscopic imaging (MRSI) by evaluating a 10-year-old boy with a history of elevated blood lead levels as a toddler (51 μg/dL at 38 months) and his 9-year-old cousin, whose blood lead levels were characterized as negligible. The MRSI technique acquires spectroscopy data simultaneously from multiple, juxtaposed regions. A reduction in NAA to Cr ratio levels for both prefrontal gray and white matter was found compared with levels in the subject’s cousin. Trope et al. (2001) further replicated this case report with 16 subjects having a history of elevated blood lead levels before 5 years of age (23–65 μg/dL). These subjects were compared with age-matched controls composed of siblings or cousins, with an average age of 8 years at time of MRSI. Control subjects had blood lead levels that never exceeded 10 μg/dL. The lead-exposed subjects exhibited a significant reduction in NAA to Cr ratio levels in frontal gray matter compared with controls. Meng et al. (2005) compared spectroscopy data obtained using the single volume element (voxel) MRS acquisition approach from children (*n* = 6) with blood lead concentrations ≥ 27 μg/dL and sex-matched control subjects (*n* = 6) with blood lead concentrations < 10 μg/dL, all approximately 11 years of age. Lead-exposed children had an average (± SD) blood concentration of 37.7 ± 5.7 μg/dL, whereas controls averaged 5.4 ± 1.5 μg/dL. Bilateral NAA, Cho, and Cr levels in the frontal lobes and hippocampi were reported as reduced in lead-exposed children relative to controls. These studies indicated disruptions in brain metabolism, particularly in the frontal lobe (Trope et al. 1998, 2001) and hippocampus (Meng et al. 2005). Because of the technical and time requirements for concentration calibration measurements in pediatric participants, many studies report results in the form of metabolite ratios instead of concentrations. These ratios are derived from integral measurements of areas underneath the individual metabolite peaks, with the Cr value serving as the denominator of the ratio and also serving as an internal reference. This may erroneously assume stability in the level of Cr. Despite their limitations, the Trope et al. and Meng et al. studies remain important, because they provided support that early childhood lead exposure alters brain metabolism.

The rationale for this current work is to determine whether childhood lead exposure permanently alters brain biochemistry with lasting effects into adulthood and to determine whether there are regional differences in brain metabolism, particularly in gray matter as implicated in the Toscano and Guilarte model. We hypothesized that adults with significant, documented childhood lead exposure would demonstrate evidence of altered brain metabolism using proton MRS via reduction of NAA. Reductions of NAA levels are thought to reflect neuronal and axonal disruption, dysfunction and/or loss consistent with brain injury.

We employed a single voxel proton MRS approach for quantification and selected a variety of gray- and white-matter structures to match established clinical deficits associated with lead exposure with corresponding regions of the brain involved with regulation of a given function. Selection of the frontal lobe locations, both medial gray and prefrontal white matter, was based upon the cognitive and behavioral features associated with lead exposure, particularly executive functioning deficits in attention, risk-taking behaviors, and inhibition (Bellinger et al. 1994; Canfield et al. 2003b, 2004; Coscia et al. 2003; Dietrich et al. 2001; Needleman et al. 2002; Nigg et al. 2010; Ris et al. 2004; Wasserman et al. 1998; Wright et al. 2008). The temporal lobe and basal ganglia voxel selection captured information for regions involved in limbic system and dopaminergic system functioning. Mood and memory functions are also implicated in these systems. Sampling within the cerebellar hemispheres and vermis was considered relevant because of the fine motor deficits and noted problems with postural sway and balance associated with lead exposure (Bhattacharya et al. 1990, 1993, 2006). The white matter of the parietal lobe was sampled, because this region is thought to be relevant for processing speed and aspects of attention. Historically, patients with lead encephalopathy have demonstrated lesions in the basal ganglia, cerebellum, cortical gray matter, and white matter (al Khayat et al. 1997; Atre et al. 2006; Mani et al. 1998; Tuzun et al. 2002).

## Materials and Methods

We have complied with all applicable requirements of the United States of America. The institutional review boards of the Cincinnati Children’s Hospital Medical Center and the University of Cincinnati approved the study protocol. A certificate of confidentiality for the study was obtained from the National Institutes of Health. All participants provided written informed consent before study participation.

### Participants

The study participants were recruited from the Cincinnati Lead Study (CLS), a longitudinal birth cohort study designed to evaluate the effects of low to moderate environmental lead exposure. The CLS enrolled pregnant women in their first or early second trimester of pregnancy who visited four prenatal clinics within impoverished Cincinnati neighborhoods with a high concentration of older, lead-contaminated housing. Women were excluded if they were known to be addicted to drugs, were diabetic, or had any known neurologic or psychiatric malady. Newborns with birth dates between 1980 and 1985 were subsequently recruited for follow-up studies. Infants were excluded if their birth weight was < 1,500 g or if genetic or other serious medical issues were present at birth. This cohort has been described periodically as cognitive, and behavioral and social assessments were completed (Dietrich et al. 1985a, 1985b, 1987a, 1987b, 1990, 1991, 1993a, 1993b, 2001). Newborns (*n* = 305) were followed up quarterly through age 5 years, semiannually from 5 to 6.5 years of age, again at 10 years, and also between the ages of 15 and 17 years. CLS subjects between 19 and 24 years of age (*n* = 194) were consecutively recruited for this MRS study via mailings, phone calls, and flyers to meet our initial sample size goal of 150 CLS participants. This size was chosen before initiating the imaging aspect of the study, because members of the CLS cohort who were pregnant, incarcerated, or claustrophobic or had metal implanted from surgeries, embedded metal from occupational exposure (e.g., welder), or gunshot wounds would not be able have a research MRI examination. Subjects who were known to be diabetic or who had any known neurologic or psychiatric illness were also excluded. The substance use of participants was determined using a clinical urine toxicology screening immunoassay for drugs of abuse, which evaluated amphetamines, barbiturates, benzodiazepine, cocaine, cannabinoids, opiates, and phencyclidine (PCP). (The only positive findings for participants were exclusively cannabinoids.) The alcohol use within the cohort was also assessed via a self-report measure previously described (Dietrich et al. 2001). Thirty-three subjects were excluded from the MRI examination because of the following: too large to fit into the MRI scanner (*n* = 8), failure to appear for the appointment (*n* = 7), pregnancy (*n* = 6), refusal due to claustrophobia (*n* = 6), nonremovable metal in their body (*n* = 5), and inability to give informed consent because of cognitive disability (*n* = 1). Two participants diagnosed with fetal alcohol syndrome as children participated in the imaging and spectroscopy session, but their data are not included in the analyses. Consistent with previous research in this cohort (e.g., Wright et al. 2008), we found no demographic or lead exposure biases associated with exclusion of these members from the analysis.

Enrollment of the participants’ mothers in the CLS permitted monitoring of blood lead levels for the participants prenatally, at birth, every 3 months to 60 months of age, and every 6 months from 60 to 78 months of age. The mean of 23 childhood blood lead assessments for each participant from 3 to 78 months of age was employed for comparison analyses. This mean childhood blood lead value has been historically used for comparisons with cognitive and behavioral outcomes (Dietrich et al. 1991, 1993b). During the first 5 years of life, at least one of the quarterly blood lead assessments exceeded 10 μg/dL for 99% of the CLS cohort. The peak lead exposure occurred between 2 and 3 years of age for the participants. By adolescence, the mean blood lead levels for the cohort were 2.8 ± 1.3 μg/dL.

### Data acquisition and processing

All MRI and MRS investigations were acquired on a 1.5 Tesla MR scanner (Signa LX; General Electric Medical Systems, Waukesha, WI, USA) with a quadrature head radiofrequency coil positioned around the head of the participant. An axial three-dimensional, inversion recovery prepped, fast spoiled gradient-echo high-resolution imaging sequence was acquired [echo time (TE) 5 msec, repetition time (TR) 12 msec, inversion time 300 msec; field of view = 24 cm × 19.2 cm, 1.5-mm thick with contiguous slices, 256 × 192 × 124 matrix for a resolution of 0.94 mm × 1 mm × 1.5 mm]. Single voxel MRS was acquired with the proton brain examination (PROBE) software (General Electric Medical Systems) using a point-resolved spectroscopy acquisition mode (PRESS) sequence (TE = 35 msec, TR = 2 sec with 64 averages) and with automatic shimming to achieve water line widths of < 5 Hz. A reference spectrum, acquired within the PROBE acquisition, collected 16 acquisitions of unsuppressed water signal for eddy current and baseline corrections. Data were processed using the LCModel program, a commercially available, user-independent, frequency domain spectral-fitting program that provides measures of metabolite concentrations (Provencher 1993). Concentration levels of NAA, Cr, Cho, mI, and GLX were determined. The concentrations are reported in institutional units (IU), rather than millimolar levels; individual corrections for magnetic field and coil variations, as well as longitudinal (T1) and transverse (T2) relaxation effects, are not accounted for because of the excessive examination times required to determine such effects. However, metabolite concentrations were adjusted for the amount of cerebrospinal fluid (CSF) in each voxel. Specifically, the LCModel software determines the metabolite concentrations assuming a parenchymal volume as defined in the MRS acquisition, typically 2 cm per voxel side for a total cubic volume of 8 cm^3^. We employed a K-means segmentation algorithm using custom software for analysis of the high resolution imaging sequence so as to determine gray matter, white matter, and CSF contributions to each voxel. We then applied a correction factor based on the ratio of volume of CSF to total parenchymal volume and adjusted each metabolite concentration accordingly. LCModel also provides a measure of data quality and reliability, reporting the Cramer–Rao lower-bound bounds (Provencher 1993). We required the metabolite Cramer-Rao value to be < 15% for inclusion in data analyses. For a given participant, regional spectroscopy data not meeting the Cramer–Rao lower-bound criteria were excluded from analyses. Approximately 98% of the > 1,100 sampled voxels of the cohort met criteria and were included in the analyses.

**Figure 1:**
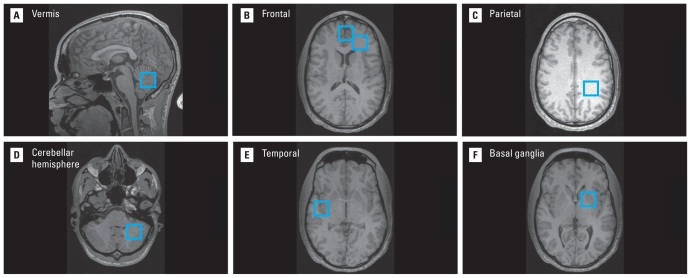
MR images with blue boxes illustrate the locations of spectroscopy voxels acquired in this study of CLS participants. The seven brain regions included (*A*) cerebellar vermis, (*B*) frontal gray matter and frontal white matter (right side of the figure), (*C*) parietal white matter, (*D*) cerebellar hemisphere, (*E*) temporal lobe at the superior temporal gyrus, and (*F*) basal ganglia. The voxels are acquired in three dimensions with a volume of 8 cc. The figure uses radiological convention for the images, where the left side of the brain appears on the right side of the image.

Seven single voxels, approximately 8 cc in volume, were positioned within the medial frontal gray matter, the left frontal white matter, left parietal white matter, the right temporal lobe at the superior temporal gyrus, the left basal ganglia, left cerebellar hemisphere (gray and white matter), and the cerebellar vermis, respectively (Figure 1). Although we anticipated no laterality differences, the left side of the brain was sampled for several reasons: *a*) neural networks associated with language function are left lateralized; *b*) most of the study population is right handed and thus likely to be left hemisphere dominant; *c*) the right hemisphere is prone to technical factors (orientation of slice-selective pulses) that produce signal artifacts (i.e., scalp lipid signal contamination) within the spectrum.

### Statistical analysis

We investigated the relationship between metabolite levels obtained from MRS analyses in seven brain regions with mean childhood blood lead level initially using Pearson and Spearman correlation coefficients. Correlation values with an alpha (*p* < 0.05) were considered significant. The metabolites examined included NAA, Cr, Cho, mI, and GLX. Recognizing that other variables could influence the association between metabolite levels and blood lead, the impact of covariates on this relationship were also examined by analyzing the bivariate relationship of the potential covariate with mean childhood blood lead level in each of the sampled brain regions, using a linear regression. Such variables that could potentially contribute included age of participant at time of imaging, Full-Scale intelligence quotient (FSIQ), current drug use (positive only for cannabinoids), sex, birth weight, and gestational age at birth. Covariates that were independently related to the outcome in one or more region at *p* < 0.10 level were included in the subsequent multiple linear regression analyses. For the linear regression analyses, beta values corresponded to the change in metabolite concentration associated with a 10-μg/dL increase in the mean childhood blood lead level and the 95% confidence intervals (CIs) determined for significance.

## Results

The CLS participants (*n* = 159) who completed the study were predominantly African American (93%), between 19 and 23 years of age, and demonstrated mean childhood blood lead values of 13.3 ± 6.1 μg/dL that ranged from 4.7 to 37.2 μg/dL (Table 1). Approximately half of the participants were male (52%). The FSIQ scores ranged from 50 to 116 with a mean of 86.8 ± 11.9. Approximately half of the participants smoked marijuana near the time of the study (48%).

Age at time of imaging and FSIQ were the only covariates meeting the criteria established *a priori* for inclusion in the model. Model estimates adjusted for age and FSIQ indicated significant inverse associations between mean childhood blood level and regional concentrations of several metabolites. Adjusted analyses estimated for a unit (micrograms per deciliter) increase in mean childhood blood lead concentrations, a decrease of NAA (β = −0.05; 95% CI, −0.1 to −0.01) and Cr (β = −0.05; 95% CI, −0.09 to −0.01) concentration levels in the basal ganglia, a decrease of NAA (β = −0.05, 95% CI, −0.11 to −0.01), and a decrease of Cho (β = −0.01; 95% CI, −0.03 to 0.00) concentration levels in the cerebellar hemisphere, a decrease of GLX (β = −0.07; 95% CI, −0.12 to −0.02) concentration levels in vermis, a decrease of Cho (β = −0.01; 95% CI, −0.01 to −0.001), and a decrease of GLX (β = −0.05; 95% CI, −0.09 to −0.01) concentration levels in parietal white matter, and a decrease of Cho (β = −0.01; 95% CI, −0.02 to −0.001) concentration levels in frontal white matter (Table 2).

We performed a secondary analysis with adjustment for age at the time of imaging only, as FSIQ may be included in the causal pathway linking lead with abnormal metabolite levels. Results of this analysis were generally comparable with those shown, except that the association between lead and NAA concentration in the cerebral hemisphere was no longer statistically significant (β = −0.05; 95% CI, −0.09 to 0.01, *p* = 0.08).

## Discussion

Our study demonstrated an inverse association between mean childhood blood lead levels and metabolic concentrations in five of the seven sampled regions. These five regions included three gray-matter regions (left basal ganglia, left cerebellar hemisphere, and the cerebellar vermis) and two white-matter regions (left frontal and left parietal).

Gray-matter sampling within the brain provided information content reflecting neuronal health. Two predominantly gray-matter regions—basal ganglia and cerebellar hemisphere—demonstrated a reduction in NAA with increased mean childhood blood lead levels. This suggests that childhood exposure to lead is associated with some level of neuronal dysfunction occurring within these regions. The diminished Cr levels associated with increased mean childhood blood lead levels further supported impaired neuronal functioning, because Cr levels are thought to reflect the status of cellular energetics. MRS studies reporting results as ratio values with Cr levels serving as an internal reference standard are likely to miss this finding.

We anticipated that NAA would inversely correlate with childhood lead exposure in the neuroanatomical regions regulating cognitive and behavioral domains found to be impaired with lead exposure, which include the frontal cortex. From a voxel-based morphometric (VBM) analysis of the volumetric imaging data, we found striking volume loss associated with mean childhood lead exposure within the medial frontal gray matter (Cecil et al. 2008). The absence of a correlation within the medial frontal gray matter was unexpected. We speculate, however, that if the neurons within the frontal cortex affected by lead suffered apoptosis, any residual cortex within this region of the brain may have viable residual neurons yielding appropriate NAA levels. This would then suggest that other sampled brain regions did not suffer significant volume loss, but dysfunction and possibly reorganization in a diminished fashion with NAA or Cho changes.

Increasing childhood mean blood lead concentrations were also correlated with GLX reductions within the cerebellar vermis as well as the parietal white matter. Although proton MRS sampled under these conditions does not provide information about glutamate specifically, the composite measure sampled with proton MRS implicates abnormal neurotransmitter functioning.

We found an inverse association between the mean childhood blood lead levels and the Cho concentration levels within the white matter of the frontal and parietal lobes and the cerebellar hemisphere, respectively. Lead can change white-matter organization and functioning via altered expression of genes essential to myelin formation (Deng and Poretz 2001; Zawia and Harry 1995), delayed differentiation of oligodendrocyte progenitors (Deng and Poretz 2001; Zawia and Harry 1995), delayed myelin accumulation (Toews et al. 1980, 1983), disordered oligodendrocyte architecture (Dabrowska-Bouta et al. 1999), structural changes within the myelin sheath and disintegration of the multilamellar structure (Dabrowska-Bouta et al. 2008), and astrogliosis (Selvin-Testa et al. 1994; Struzynska et al. 2001, 2007).

The VBM analyses (Brubaker et al. 2010; Cecil et al. 2008) for this same cohort did not show any lead-associated changes in white-matter volumes. Reductions of Cho concentration levels within white-matter regions of the frontal and parietal lobes and the cerebral hemisphere were observed without any NAA-correlated changes. The Cho signal observed on proton MRS arises primarily from the mobile, intracellular pools of phosphocholines and glycerophosphocholines involved in membrane synthesis and degradation (Cecil and Jones 2001; Stork and Renshaw 2005). Phosphatidylcholine head groups from myelin are not MRS visible, because their relaxation times are too short for signal detection. Typically, Cho increases on proton MRS characterize neoplastic or demyelinating pathologies. A finding of decreased Cho concentration level is unusual, although it has been reported in hypomyelination disorders (van der Voorn et al. 2006). It may represent abnormal chemical composition within the architecture of myelin. A recent diffusion tensor imaging analysis of 91 representative members of the CLS cohort revealed lead-associated reductions in fractional anisotropy (FA) within the white matter (Brubaker et al. 2009). Further interrogation found that the changes in FA could be attributed to significant changes in radial diffusivity (RD). RD primarily reflects alterations in the myelin sheath thickness and organizational characteristics. The lead-associated declines in RD and the reduction in Cho are consistent with abnormal myelin architecture affecting white matter organization.

The beta values associated with a 1-μg/dL increase in mean childhood blood lead levels for metabolite concentrations are small, which is physiologically expected. An MRS profile for a brain tumor is typically quite distinct from a region from an acute stroke. In these conditions, metabolite levels dramatically change several orders of magnitude because of the macroscopically observed changes to the brain. For conditions such as low-level lead exposure, where the brain appears relatively normal without lesions, the model of psychiatric diseases such as schizophrenia and bipolar disorder can be considered. These conditions can be clinically diagnosed with high degrees of certainty. However, if a person with a diagnosis of a given psychiatric condition has imaging or spectroscopy performed in a blinded fashion, it is likely to be interpreted within normal limits because of the lack of diagnostic features attributed to the conditions and broad normative values in a clinical setting. In contrast, research studies of such psychiatric conditions compared with appropriately screened and matched healthy control participants can find abnormal metabolite levels associated with a psychiatric condition. Given that the historical blood lead levels of an age- and demographically matched control population would not be known from childhood, nor would the blood lead levels for a young adult be zero, ideal comparison studies with the CLS population are not possible outside of the regression approach applied within this study.

### Limitations

The lack of correlative findings within the temporal lobe may arise from technical factors associated with the proton MRS acquisition. Peak broadening due to magnetic susceptibility and field inhomogeneity arises at the interface between the temporal lobe and adjacent structures, including the ear and the temporal bone, resulting in incomplete water suppression, broad line widths of the metabolites, and failure of metabolite quantification. A dedicated MRS examination optimizing the homogeneity of the temporal lobe is necessary to clarify the impact of lead exposure within the temporal lobe. A dedicated study of the hippocampus is also warranted from other reports in the literature providing experimental evidence.

## Conclusions

The neurochemical changes demonstrated in the adult participants of the CLS reflect the complexities of lead neurotoxicity. Gray-matter reduction of NAA with the basal ganglia and cerebellum is consistent with the concept that sustained childhood lead exposure produces an irreversible pattern of neuronal dysfunction. White-matter choline changes within the frontal and parietal lobes suggest a permanent alteration to myelin architecture.

## Figures and Tables

**Table 1:** Relevant participant characteristics from the CLS included in the MR spectroscopy study.

Characteristic	Values
Age [years (mean ± SD)]	20.8 ± 0.91
Range (years)	19.7–22.9
Race [*n* (%)]	
Black	147 (93)
White	11 (6.9)
Biracial	1 (0.1)
Sex [*n* (%)]	
Female	76 (48)
Male	83 (52)
Mean childhood blood lead level [μg/dL (mean ± SD)]	13.3 ± 6.1
Range (μg/dL)	4.65–37.2
Gestational age at birth [weeks (mean ± SD)]	39.4 ± 1.7
Range (weeks)	35–43
Birth weight [g (mean ± SD)]	3,106 ± 467
Range (g)	1,814–4,260
IQ-FSIQ (at 7 years)	86.8 ± 11.9
Range	50–116
Marijuana use [*n* (%)]	
Positive	76 (48)
Negative	83 (52)

**Table 2:** Regression coefficients between mean childhood blood lead and measured regional metabolite concentration levels in an unadjusted model and in a model adjusted for age at time of imaging and FSIQ, respectively.

		Unadjusted		Age and FSIQ adjusted	
Voxel location	Metabolite	Beta	95% CI	Beta	95% CI
Basal ganglia (left)	NAA	−0.05	−0.09 to −0.003	−0.05	−0.1 to −0.01*
	Cr	−0.05	−0.08 to −0.01	−0.05	−0.09 to −0.01*
	Cho	−0.01	−0.02 to 0.001	−0.01	−0.02 to 0.002
	GLX	−0.07	−0.16 to 0.02	−0.08	−0.18 to 0.01
	mI	0.001	−0.03 to 0.03	0.001	−0.03 to 0.04

Cerebellar hemisphere (left)	NAA	−0.06	−0.11 to −0.01	−0.05	−0.11 to −0.01*
	Cr	−0.02	−0.06 to 0.02	−0.02	−0.06 to 0.02
	Cho	−0.01	−0.03 to 0.00	−0.01	−0.03 to 0.00*
	GLX	−0.19	0.02 to 0.01	−0.07	−0.15 to 0.01
	mI	−0.02	−0.07 to 0.02	−0.02	−0.06 to 0.03

Cerebellar vermis (midline)	NAA	−0.02	−0.05 to 0.01	−0.01	−0.04 to 0.01
	Cr	−0.02	−0.05 to 0.01	−0.01	−0.04 to 0.01
	Cho	−0.005	−0.01 to 0.002	−0.005	−0.01 to 0.003
	GLX	−0.06	−0.11 to −0.01	−0.07	−0.12 to −0.02*
	mI	−0.001	−0.03 to 0.03	0.01	−0.02 to 0.04

Frontal gray matter (medial)	NAA	−0.02	−0.04 to 0.01	−0.02	−0.04 to 0.01
	Cr	−0.01	−0.03 to 0.01	−0.01	−0.03 to 0.01
	Cho	−0.004	0.01 to 0.002	−0.003	−0.01 to 0.004
	GLX	−0.03	−0.08 to 0.02	−0.02	−0.08 to 0.03
	mI	0.02	−0.01 to 0.04	0.02	−0.01 to 0.04

Frontal white matter (left)	NAA	−0.02	−0.05 to 0.01	−0.02	−0.05 to 0.02
	Cr	−0.004	−0.03 to 0.02	−0.004	−0.03 to 0.02
	Cho	−0.01	−0.02 to −0.004	−0.01	−0.02 to −0.001*
	GLX	−0.08	−0.15 to −0.004	−0.06	−0.14 to 0.01
	mI	0.003	−0.02 to 0.03	0.004	−0.03 to 0.03

Parietal white matter (left)	NAA	−0.02	−0.05 to 0.004	0	−0.04 to 0.01
	Cr	−0.01	−0.03 to 0.003	−0.01	−0.02 to 0.01
	Cho	−0.01	−0.01 to −0.002	−0.01	−0.01 to −0.001*
	GLX	−0.05	−0.09 to −0.01	−0.05	−0.09 to −0.01*
	mI	0	−0.03 to 0.02	−0.001	−0.02 to 0.02

Temporal lobe (STG-left)	NAA	−0.02	−0.07 to 0.02	−0.02	−0.06 to 0.03
	Cr	−0.01	−0.04 to 0.01	−0.01	−0.04 to 0.02
	Cho	−0.01	−0.01 to 0.002	−0.01	−0.01 to 0.002
	GLX	−0.03	−0.09 to 0.03	−0.03	−0.09 to 0.04
	mI	−0.02	−0.05 to 0.01	−0.01	−0.04 to 0.02

* *p* < 0.05.
